# ABA Metabolism and Homeostasis in Seed Dormancy and Germination

**DOI:** 10.3390/ijms22105069

**Published:** 2021-05-11

**Authors:** Naoto Sano, Annie Marion-Poll

**Affiliations:** IJPB Institut Jean-Pierre Bourgin, INRAE, AgroParisTech, Université Paris-Saclay, 78000 Versailles, France; naoto.sano@inrae.fr

**Keywords:** abscisic acid, biosynthesis, catabolism, dormancy, germination, natural variation, seed, transcription factor

## Abstract

Abscisic acid (ABA) is a key hormone that promotes dormancy during seed development on the mother plant and after seed dispersal participates in the control of dormancy release and germination in response to environmental signals. The modulation of ABA endogenous levels is largely achieved by fine-tuning, in the different seed tissues, hormone synthesis by cleavage of carotenoid precursors and inactivation by 8′-hydroxylation. In this review, we provide an overview of the current knowledge on ABA metabolism in developing and germinating seeds; notably, how environmental signals such as light, temperature and nitrate control seed dormancy through the adjustment of hormone levels. A number of regulatory factors have been recently identified which functional relationships with major transcription factors, such as ABA INSENSITIVE3 (ABI3), ABI4 and ABI5, have an essential role in the control of seed ABA levels. The increasing importance of epigenetic mechanisms in the regulation of ABA metabolism gene expression is also described. In the last section, we give an overview of natural variations of ABA metabolism genes and their effects on seed germination, which could be useful both in future studies to better understand the regulation of ABA metabolism and to identify candidates as breeding materials for improving germination properties.

## 1. Introduction

During seed maturation, abscisic acid (ABA) induces an adaptive trait called primary dormancy that prevents vivipary and, after seed dispersal, delays and spreads germination over time. Exogenous signals, especially temperature variations during seed set, have been described to have a major effect on the modulation of dormancy depth [1]. The dormancy depth determines the timing of germination under seasonally varying conditions at which seedling survival and growth are the most favorable [2]. Seed dormancy is often a combination of a coat-imposed dormancy due to the multiple cell layers surrounding the embryo (testa and endosperm) preventing the radicle protrusion, and embryonic dormancy, when the embryo itself is unable to induce growth [3,4]. After seed shedding, dormancy is released upon imbibition by ABA degradation, which precedes the activation of germination by a second plant hormone, gibberellin (GA). The balance between these two hormones has been shown to integrate light, temperature or nitrate signals and act antagonistically on embryo growth and endosperm weakening [5,6,7,8]. Storage or imbibition of non-dormant seeds in unfavorable conditions for germination can trigger a secondary dormancy. This is a way to prevent seeds from germinating in an untimely fashion while inducing a seasonal cycling of dormancy level in seeds [9,10].

Significant efforts have been made over the last decades to understand the ABA signaling mechanisms controlling dormancy and germination. In seeds as in vegetative tissues, ABA perception involves a multigene family encoding pyrabactin resistance/PYR-like/regulatory components of ABA receptor (PYR/PYL/RCAR), which sequester and inhibit protein phosphatases 2C (PP2C), when ABA is present. PP2C inactivation allows phosphorylation of SNF1-related kinases 2 (SnRK2), which in turn phosphorylate bZIP transcription factors of the ABI5/AREB/ABF family, which bind to ABA response elements (ABRE) in promoter sequences of ABA-inducible genes [11]. The germination phenotypes of PYR/PYL/RCAR, PP2C or SnRK2 multiple mutants suggest that the PYR/PP2C/SnRK2 phosphorylation cascade operates in dormancy regulation [12,13]. Moreover, recent studies reported the interaction of DELAY OF DORMANCY (DOG1), a major regulator of seed dormancy, for which molecular function remains elusive, with a subset of clade A PP2C phosphatases, resulting in their inhibition [14,15,16].

Research on the metabolic pathway of ABA has a long history, and its relationship with germination and dormancy has been broadly explored [17,18,19,20]. Nevertheless, novel ABA metabolism-related genes and factors involved in their regulation have been discovered in recent years. In this review, we first summarize current knowledge about the molecular characterization of genes of the ABA metabolism pathway. How the ABA metabolism-related genes are involved in the induction of dormancy during seed development, as well as in the dormancy release and germination after imbibition is then discussed, with particular emphasis on specific regulatory factors that fine-tune ABA levels, modulate dormancy depth and control germination, in response to major environmental cues. Finally, we outline findings on the natural variations of genes affecting ABA metabolism in seeds and summarize how these variations affect ABA metabolism and seed germination for a given species.

## 2. An Overview of the ABA Metabolism Pathway

### 2.1. Carotenoid Interconversion and Cleavage in Plastids

**Figure 1:**
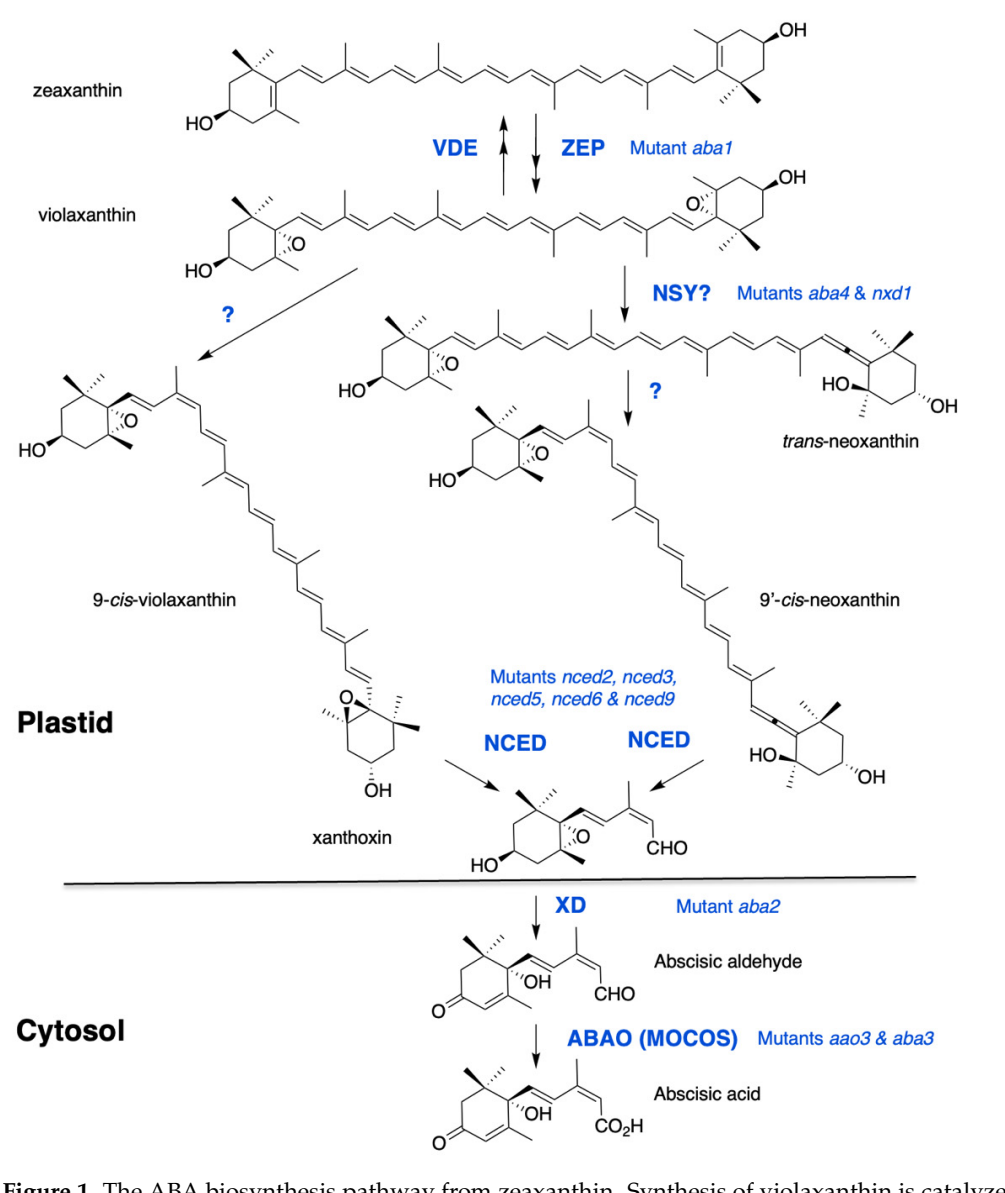
The ABA biosynthesis pathway from zeaxanthin. Synthesis of violaxanthin is catalyzed by zeaxanthin epoxidase (ZEP). A reverse reaction catalyzed by violaxanthin de-epoxidase (VDE) contributes to energy dissipation in chloroplasts under high light. The formation of *cis*-isomers of violaxanthin and neoxanthin remains elusive. Two proteins, ABA-DEFICIENT4 and NEOXANTHIN-DEFICIENT1, are necessary for neoxanthin synthase (NSY) activity. Recent evidence suggests that ABA4 is also involved in ABA synthesis from 9-*cis*-violaxanthin. Cleavage of *cis*-xanthophylls is catalysed by a family of 9-*cis*-epoxycarotenoid dioxygenases (NCED). Xanthoxin is then converted by a xanthoxin dehydrogenase (XD) into abscisic aldehyde, which is oxidized into ABA by an abscisic aldehyde oxidase (ABAO). ABAO contains a molydenum cofactor activated by a MoCo sulfurase (MOCOS). Arabidopsis mutants are indicated for each enzymatic step.

ABA is a sesquiterpenoid that is synthesized in plants via the cleavage of C_40_ carotenoid precursors. The early steps of ABA biosynthesis take place in plastids and the direct precursors are oxygenated carotenoids, also called xanthophylls. The first carotenoid, *cis*-phytoene, derives from a C_5_ precursor, common to all isoprenoids, isopentenyl diphosphate (IPP). Successive desaturation and isomerization reactions lead to the formation of all-*trans*-lycopene, in which β-cyclization gives rise to β-carotene. Zeaxanthin is produced by the hydroxylation of β-carotene and this first xanthophyll is often chosen as the starting point of the ABA biosynthesis pathway, as shown in Figure 1. Indeed, the first *Arabidopsis thaliana* mutants identified on the basis of their ABA-deficient phenotypes were affected in zeaxanthin epoxidation; nevertheless, carotenoid cleavage is now considered to be the first committed step in ABA synthesis [21].

Zeaxanthin is converted by zeaxanthin epoxidase (ZEP) to violaxanthin via the intermediate antheraxanthin. Mutants defective in zeaxanthin epoxidation were selected on germination screens and their ABA deficiency resulted in reduced seed dormancy and hypersensitivity to water stress in both Arabidopsis and *Nicotiana plumbaginifolia* [22,23,24]. ZEP proteins are flavin-dependent monooxygenases that catalyze the introduction of molecular oxygen into zeaxanthin [25,26]. The reverse reactions, from violaxanthin to zeaxanthin, and through antheraxanthin, are catalyzed by violaxanthin epoxidase (VDE). These two proteins share a lipocalin domain involved in lipophilic substrate binding and the so-called xanthophyll cycle has a protective role against excessive light [27]. No correlation between VDE regulation and ABA accumulation has been reported [28].

Violaxanthin is formed as an all-*trans*-isomer and can be either isomerized into 9-*cis*-violaxanthin or converted into the all-*trans*-isomer of neoxanthin (*trans*-neoxanthin), which is then isomerized into 9′-*cis*-neoxanthin. Both 9-*cis*-violaxanthin and 9′-*cis*-neoxanthin can be cleaved in vitro into the first C_15_ precursor of ABA, xanthoxin [29]. Despite recent evidence, the enzymatic steps leading to the formation of these last *cis*-xanthophyll precursors of ABA still remain elusive. Two genes have been identified, *ABA-DEFICIENT4* (*ABA4*) and *NEOXANTHIN-DEFICIENT1* (*NXD1*), whose lack-of-function prevents neoxanthin synthesis. The Arabidopsis *aba4* mutant was isolated in a screen for germination resistance to paclobutrazol, which is an inhibitor of GA synthesis [30]. The mutant *nxd1* was identified in tomato (*Solanum lycopersicon*) based on its paler yellow flower color [31]. In both mutants, violaxanthin accumulation was increased compared to wild type and both 9′-*cis* and *trans*-neoxanthin isomers were absent, in contrast to 9-*cis*-violaxanthin, which was still present. Intriguingly, despite their similar carotenoid defects, these mutants showed opposite ABA-related phenotypes. Notably, in Arabidopsis *aba4*, ABA levels were significantly reduced in both seeds and water-stressed rosettes [30], while in tomato *nxd1* no ABA deficiency was observed [31]. A recent genetic analysis confirmed that, in Arabidopsis also, *nxd1* alleles were not ABA-deficient and even overproduced ABA compared to wild type, likely due to an increased 9-*cis*-violaxanthin production in the absence of neoxanthin synthesis. In agreement, *nxd1* mutants exhibited increased seed dormancy and water stress tolerance. More importantly, the *aba4* mutation was shown to be epistatic to *nxd1*, suggesting that *aba4* prevented ABA production from both 9-*cis*-violaxanthin and 9′-*cis*-neoxanthin [32]. Moreover, overexpression of ABA4 in both wild-type and *nxd1* backgrounds increased 9-*cis*-violaxanthin and ABA accumulation [30,32]. Therefore, ABA4 has been postulated to contribute to *trans* to *cis* violaxanthin isomerase activity, producing both *cis*-violaxanthin and neoxanthin precursors. Since NXD1 is a cytosolic protein which likely cannot interact with ABA4 in plastids, it has been further hypothesized that NXD1 possibly produces an unknown factor required for neoxanthin formation, but is not necessary for that of 9-*cis*-violaxanthin [32]. Further biochemical evidence would be essential to confirm the hypothetical functions of ABA4 and NXD1 and identify putative cofactors.

The first dedicated step to ABA biosynthesis is the cleavage of *cis*-isomers of violaxanthin and neoxanthin, by a 9-*cis* epoxycarotenoid dioxygenase (NCED). The first *NCED* gene, *VIVIPAROUS14,* was cloned in maize (*Zea mays*) after the isolation of the viviparous ABA-deficient mutant *vp14* [33]. The VP14 protein is a non-heme iron-dependent dioxygenase that cleaves only 9-*cis*-epoxy-xanthophyll isomers at the 11–12 position [34]. In every plant species examined, *NCED* genes belong to a multigene family and in Arabidopsis, NCED is encoded by a family of 5 members namely, *NCED2*, *NCED3*, *NCED5*, *NCED6* and *NCED9* [35] and belongs to a larger family of 9 members, which includes other carotenoid cleavage dioxygenases (CCD1, CCD4, CCD7 and CCD8) having different substrate specificities [36]. Maize VP14 and Arabidopsis NCED3 have been described to exist in two forms—a smaller one, which is soluble in the stroma, and a larger one bound to thylakoid membranes. VP14 and all five NCEDs exhibit similar N-terminal amphipathic helices that are required for attachment to the thylakoid membrane [37,38,39,40]. In contrast to NCED, ZEP and ABA4 are found to be associated with the plastid inner envelope. Furthermore, ABA4 sequence contains predicted transmembrane domains, indicating that it might be an integral protein [30,41]. However, translocation mechanisms of these lipophilic metabolites within plastids remain to be explored. Carotenoid cleavage in plastids produces xanthoxin, which has to move to the cytosol before conversion into ABA, but no transporter has yet been described.

### 2.2. Cytosolic Steps from Xanthoxin to ABA

Abscisic aldehyde is synthesized from xanthoxin by an enzyme belonging to the family of short-chain dehydrogenases/reductases (SDR). The first mutant *aba2-1* was isolated in Arabidopsis, based on its capacity to germinate in the presence of paclobutrazol, an inhibitor of gibberellin biosynthesis [42,43]. It exhibited very severe phenotypes, suggesting the absence of ABA2 functional redundancy with other SDR family members. The *ABA2* locus, also named SDR1, encodes a cytosolic NAD-dependent oxidoreductase with xanthoxin dehydrogenase (XD) activity [44,45]. Intragenic complementation of mutant alleles has been observed, suggesting that ABA2 may have a multimeric structure [46,47].

The oxidation of the abscisic aldehyde to the carboxylic acid is the final step in ABA biosynthesis, which is catalyzed by an abscisic aldehyde oxidase. Among four Arabidopsis aldehyde oxidases (AAOs), AAO3 has been shown to be active on abscisic aldehyde [48,49]. In accordance, *aao3* alleles exhibited ABA-related phenotypes including the expected reduction in ABA content, water stress tolerance and seed dormancy [50]. AAO3 is a molybdoenzyme that requires a molybdenum cofactor (Moco) for catalytic activity. Mutations in genes for MoCo biosynthesis induce defects in a number of molybdoenzymes inducing pleiotropic phenotypes besides ABA deficiency. Mutants affected in the last step of MoCo biosynthesis have been identified on their ABA-deficient phenotypes and named *aba3* in Arabidopsis. ABA3 encodes a Moco sulfurase (MOCOS), which provides the terminal sulfur ligand in Moco [51,52].

### 2.3. ABA Conjugation and Hydroxylation

**Figure 2:**
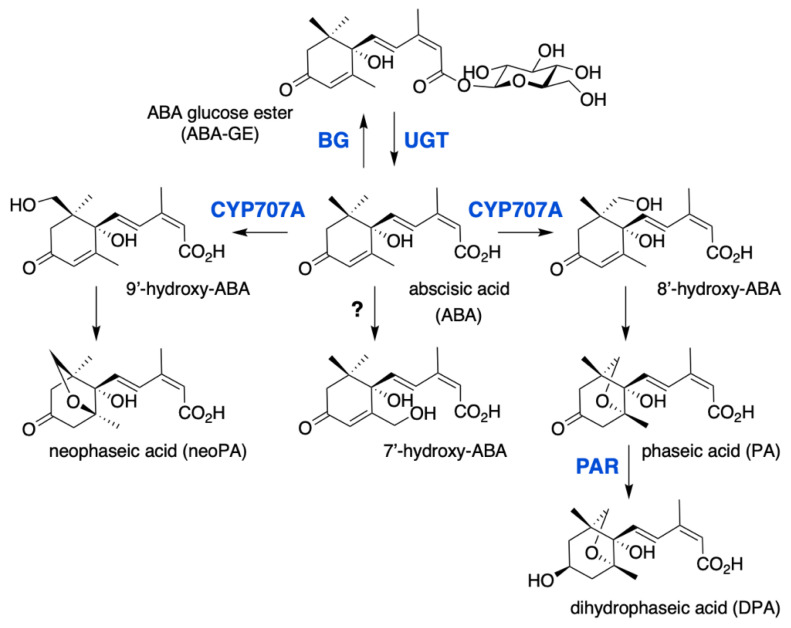
ABA catabolic pathways. ABA 8′-hydroxylase is encoded by the *CYP707A* gene family and converts ABA into 8′-hydroxy-ABA, which undergoes a spontaneous isomerization to give phaseic acid (PA). PA reductase (PAR) then converts PA into dihydrophaseic acid (DPA). 7′ and 9′ hydroxylations are minor catabolic routes, 9′-hydroxy-ABA is produced by CYP707A and converted into neoPA, but the enzyme responsible for 7′-hydroxylation remains unknown. ABA is also inactivated to ABA glucose ester (ABA-GE) by UDP-glucosyltransferases (UGT), ABA-GE is then converted to free ABA by β-glucosidases (BG).

Active hormone levels are modulated by degradation or conjugation (Figure 2). The major catabolic route is the 8′-hydroxylation of ABA by the CYP707A subfamily of P450 monooxygenases [53,54], which are also called ABA 8′-hydroxylases (ABA8′OH or ABA8′ox). In Arabidopsis, this enzyme is encoded by a gene family of four members, *CYP707A1* to *A4*, and multiple mutants were shown to accumulate large ABA amounts and exhibit a very strong seed dormancy [55]. ABA hydroxylation can also occur at the C-7′ and C-9′ position and 9′-hydroxylation is catalyzed by CYP707As as a side reaction, in contrast to C-7′ which hydroxylation mechanism remains unknown. Spontaneous isomerization of 8′- and 9′-hydroxy-ABA gives rise respectively to phaseic acid (PA) and neoPA [56,57]. PA is then converted to dihydrophaseic acid (DPA) by the PA reductase (PAR) which belongs to the family of dihydroflavonol 4-reductase (DFR)-like NAD(P)H-dependent reductases [58]. Moreover, Weng et al. reported that PA was able to activate a subset of ABA-responsive genes and bind to members of the ABA receptor family [58]. Thus, despite less effective than ABA, PA likely has residual biological activity, in contrast to DPA which is inactive.

The conjugation of ABA with glucose to form the ABA-glucose ester (ABA-GE) is catalyzed by uridine diphosphate glucosyl transferases (UGTs), which belong to the superfamily of glycosyltransferases. Eight Arabidopsis UGTs have been shown to have in vitro activity on ABA, however only UGT71B6 exhibits a selective glucosylation activity towards the natural enantiomer (+)-ABA [59,60]. Gene overexpression or inactivation by either mutation or RNA interference has suggested a biological role for UGT71B6 and its closest homologs, UGT71B7 and UGT71B8, UGT71C5 and UGT75B1 in ABA conjugation in planta [60,61,62,63]. In contrast to hydroxylation that leads to the irreversible degradation of ABA, conjugation to glucose produces a storage form of the hormone that can be converted back to ABA by glucosidases (BGs). In Arabidopsis, two glucosidases BG1 (BGLU18) and BG2 (BGLU33), localized respectively in the endoplasmic reticulum or the vacuole, have been shown to catalyze ABA-GE hydrolysis [64,65].

## 3. ABA Metabolism and Seed Dormancy Induction

### 3.1. Control of ABA Levels by Carotenoid Cleavage and ABA Hydroxylation

During seed maturation, ABA positively regulates reserve accumulation, inhibits embryo growth and induces desiccation tolerance and primary dormancy. ABA accumulated in seeds originates from synthesis in both maternal and zygotic tissues and transport from vegetative tissues; however, only ABA produced by zygotic tissues during late maturation stages has a predominant role in dormancy induction [66,67,68,69]. Carotenoid cleavage by NCED and ABA inactivation by CYP707A 8′-hydroxylase have been proven to constitute key regulatory steps for the control of ABA accumulation. Among the five Arabidopsis *NCED* genes, *NCED6* exhibits the highest expression levels in developing seeds and is specifically expressed in the endosperm. *NCED9* expression has been detected in testa at early stages and in embryo at later stages. *NCED5* is expressed at lower levels, as compared to *NCED6* and *NCED9*, but its tissue specificity is broader in both embryo and endosperm all along seed development. Mutant analysis indicated that expression of these three genes in both embryo and endosperm makes a major contribution to ABA production for dormancy induction [70,71]. Moreover, the comparison of ABA levels in mutants *nced2569* and *nced259* has suggested that NCED6 activity in endosperm is responsible for most of the xanthoxin production that gives rise to ABA in developing seeds [72]. As in Arabidopsis, ABA levels in barley (*Hordeum vulgare*) seeds peak at mid-development and ABA abundance is correlated with an increase in *HvNCED2* expression [73]. In maize, *VP14* transcript has been detected in embryo and its function is required in dormancy induction since gene mutation leads to a viviparous phenotype [74].

ABA catabolism has a great role in the regulation of active hormone levels during seed development. High levels of catabolites, mainly DPA, have been detected in Arabidopsis developing siliques and seeds [55,68,72]. Furthermore, in *cyp707a* mutants, enhanced dormancy and ABA accumulation has further proven the important contribution of *CYP707A1* and *CYP707A2*, which transcripts are highly accumulated at early and late maturation stages respectively [55]. Maximal accumulation of ABA and DPA is observed at mid-development stages. The higher abundance of DPA relatively to ABA reinforces the importance of inactivation of hormone pools to maintain hormone homeostasis. While ABA production in early stages is essential for embryo development and growth arrest, a too-high seed content at later stages can lead to excessive dormancy depth. Interestingly, a coordinated regulation among catabolic pathways is likely occurring in developing seeds to prevent ABA excess. In *cyp707a1a2* double mutants, absence of 8′-hydroxylation can be partially compensated by the production of high amounts of ABA-GE and 7′-OH-ABA [72]. In cereals, ABA catabolism by CYP707A family members has also been described to regulate ABA levels in developing seeds [7]. In barley, contrasting with *HvNCED2* which is responsible for ABA accumulation at early and middle stages, *HvABA’OH1* has been reported to control ABA levels at later maturation [73]. Furthermore, in wheat (*Triticum aestivum*), mutations affecting *TaABA8′OH1* expression were shown to result in higher ABA content in embryos during seed development, in correlation with a lower germination in the field [75].

### 3.2. Influence of Maternal Environmental Conditions on Dormancy and Germination

Environmental conditions during seed set modulate dormancy depth and then influence dormancy release and germination after seed dispersal. Temperature, light and soil nitrate conditions experienced by the mother plant during seed development are important signals that regulates dormancy and germination vigour. As reported in a number of species, maternal temperature variations have the greatest effect on dormancy and ABA content in seed progeny. In Arabidopsis, the enhancement of dormancy by cold temperature is correlated with increased ABA and decreased *CYP707A2* transcript levels in dry seeds [76]. In wheat, seeds developed under high or low temperatures exhibit either weak or strong dormancy, respectively; however, dormancy depth does not seem to be strictly associated with modifications in ABA content in dry seeds. As reported in a recent study, no differences in ABA contents were observed in dry seeds produced under either low (13 °C) or high (28 °C) temperature. However, the stronger dormancy that was induced by low maternal temperature was associated with higher ABA levels after 24 h imbibition and modulation of the expression of *TaNCED1*, *TaNCED2* and *TaCYP707A1* during imbibition [77]. Conversely, in oilseed rape (*Brassica napus*), seeds produced under elevated temperatures show reduced ABA contents and high pre-harvest sprouting rates [78]. In rice (*Oryza sativa*), heat stress during seed filling has been described to delay germination even after dormancy release treatment and this delay is correlated with an increased expression of *NCED* genes and a decreased expression of *CYP707A* genes in imbibed seeds. Hot temperatures have been also shown to affect methylation of catabolism gene promoters and therefore have been suggested to influence catabolism gene expression, ABA levels and germination rates [79].

High nitrate feeding of Arabidopsis wild-type plants increase endogenous nitrate content in mature dry seeds, which is correlated with the production of less dormant seeds, whereas low nitrate has the opposite effect [18,80,81,82]. In accordance with the reduction of dormancy depth, lower ABA levels are observed in seeds produced under high nitrate conditions. As under cool temperature, the higher expression of the *CYP707A2* gene at late maturation stages suggested a possible role for *CYP707A2* in controlling seed ABA levels in response to endogenous nitrate [83,84]. Functional genomics analysis of seeds matured under low temperature or low nitrate showed that the similar effects of temperature and nitrate on seed performance, and notably seed dormancy, were reflected by partly overlapping genetic and metabolic networks [85].

Light intensity and spectral quality during seed development can alter dormancy depth, as reviewed by Chahtane et al. [6]. Low light intensity or high ratio of far red (FR) to red (R) have been described to result in deeper dormancy in Arabidopsis seeds. However, light responses may depend on species and culture conditions. Recently, the effect of light quality has been assessed on seeds harvested from soybean (*Glycine max*) plants grown in the field [86]. The shading of the mother plant on germination of soybean seeds was analyzed in comparison to that of normal light, in two different field culture conditions, either maize-soybean intercropping or soybean monocropping culture. The shade environment, which results in a higher FR/R ratio and a lower photon flux, promoted seed germination and decreased ABA content in both dry and imbibed seeds. In accordance, the expression of the ABA biosynthesis gene *ABA2* was decreased while that of the catabolism gene *CYP707A1* was increased.

### 3.3. Regulatory Factors of ABA Metabolism in Developing Seeds

The LAFL proteins, LEAFY COTYLEDON 2 (LEC2), ABSCISIC ACID INSENSTIVE 3 (ABI3), and FUSCA3 (FUS3) B3 domain transcription factors, together with LEC1 and LEC1-LIKE (L1L), homologs of the NF-YB subunit of the CCAAT-binding complex are major regulators of seed development, which control embryo growth arrest, reserve storage and dormancy induction [87]. Mutants in these genes share some common phenotypes, such as a reduced accumulation of storage compounds, desiccation intolerance and precocious germination [88]. Furthermore, LAFL genes have been reported to interact with hormone signaling and synthesis pathways to regulate seed development and germination processes. During seed development, FUS3 promotes ABA accumulation and inhibits GA biosynthesis, thus contributing to dormancy induction [89]. However, while FUS3 directly acts on GA biosynthesis gene expression, its control on ABA pathway may be indirect since no targets have been identified.

**Figure 3:**
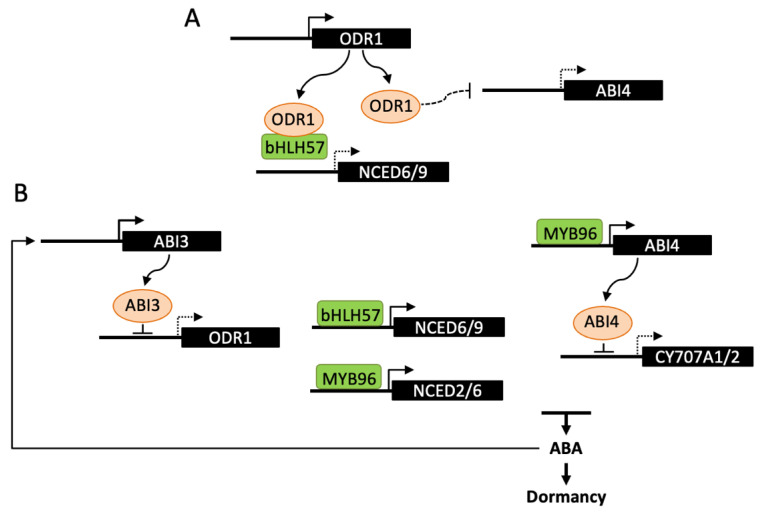
Transcriptional regulation of ABA metabolism in dormancy induction. The transcription factors ABI4, MYB96 and bHLH57 have been described to directly bind promoters of either *NCED* or *CYP707A* genes and regulate ABA levels and dormancy depth. (**A**) ODR1 interaction with bHLH57 prevents binding to *NCED6* and *NCED9* promoters and activation of ABA biosynthesis. ODR1 may also regulate ABA levels by decreasing the transcription of *ABI4*. (**B**) During seed maturation, ABI3 binding to the *ODR1* promoter represses its expression and releases bHLH57 inhibition thus promoting ABA biosynthesis and seed dormancy. This regulation may be amplified by the stimulation of ABI3 by ABA. *ABI4* has been shown to bind *CYP707A1/2* promoters and inhibit ABA catabolism, leading to higher ABA levels and seed dormancy. MYB96 would have a dual function in the regulation ABA metabolism by directly activating *NCED2* and *NCED6* and indirectly repressing *CYP707A2* through its positive effect on *ABI4* expression. Dashed lines indicate either an indirect effect of the signaling factor, or a reduced expression of the downstream gene.

ABI3 is a central transcription factor in the ABA signaling network and, in contrast to other *lafl*, Arabidopsis *abi3* mutants have been identified by their germination insensitivity to ABA [90]. ABI3 has been recently reported to bind to the promoter and repress the expression of *REVERSAL of RDO5 1* gene (*ODR1*) and ODR1 to act as a regulator of ABA biosynthesis [91]. *DELAY OF GERMINATION18* (*DOG18*)/*REDUCED DORMANCY 5* (*RDO5*) was previously identified by both mutant screen and QTL analysis for low dormancy. *RDO5* encodes a PP2C pseudophosphatase, that does not belong to the clade A, which includes PP2Cs interacting with ABA receptors [92]. The mutant *odr1* was selected in a suppressor screen after *rdo5* mutagenesis and *odr1* seeds exhibit increased ABA levels and dormancy, in both *rdo5* and wild-type backgrounds. *ODR1* encodes a homolog of rice *Seed dormancy 4* (*Sdr4*), which is a zinc finger protein identified by QTL cloning [93]. In Arabidopsis, ODR1 was shown to interact with bHLH57, a basic helix-loop-helix transcription factor, which binds to *NCED6* and *NCED9* promoters and activates gene expression (Figure 3). ODR1 protein itself cannot directly bind to *NCED* promoters, but its interaction with bHLH57 in a complex negatively impacts on *NCED* gene transcription and consequently on dormancy induction [91]. In contrast to ODR1, the expression of the rice Sdr4 protein is positively regulated by OsVP1, the rice ortholog of ABI3, and in accordance positively affects dormancy in several rice cultivars [93]. The opposite effects of ODR1 and Sdr4 on seed dormancy may result from their differential regulation by either ABI3 or VP1 and also from differences in expression of Arabidopsis and rice *DOG1* homologs, *DOG1* being upregulated in *odr1* and downregulated in *sdr4*. The regulation of ABA metabolism by rice Sdr4 still remains to be studied [91]. Together with *DOG1*, expression of the genes encoding ABI4 and the Drought Response Element binding protein DREB2C is increased in *odr1* dry and hydrated seeds, compared to wild-type, however no direct protein interaction with ODR1 has been observed. Since *DREB2C* and *ABI4* transcription factors have been previously shown to interact with *NCED6* and *NCED9* promoters, ODR1 has been hypothesized to act not only through direct interaction with bHLH57, but also indirectly by downregulating the expression of *ABI4* and *DREB2C* [91,94,95].

ABI4 and DREB2C belong to the family of APETALA2/ethylene responsive element binding protein (AP2/EREBP)-like transcription factors. In contrast to DREB2C which is one of five members of the clade A2, ABI4 is the single member of the clade A3, suggesting absence of functional redundancy with other family members [96]. As described below (Section 4.3), both have been reported to regulate ABA biosynthesis during seed germination [94,97]. Nevertheless, further evidence suggested an additional role for ABI4 in the regulation of hormone levels during seed development [97]. Like ABI3, ABI4 has been identified based on ABA-insensitive germination of mutants. However, it has been later shown to be involved not only in ABA signaling and germination, but also in a number of other signaling pathways and physiological processes [98]. In *abi4* alleles, a mildly reduced dormancy of dry seeds is observed, moreover seeds inside immature green siliques exhibit a viviparous phenotype when siliques are detached and plated on soil or medium. These phenotypes have been correlated with decreased ABA levels and increased *CYP707A* gene expression in dry seeds. Since ABI4 is able to bind *CYP707A1* and *A2* promoters, it has been suggested that ABI4 positively regulates dormancy induction by repressing ABA catabolism [97]. The R2R3-type MYB transcription factor MYB96 has also been shown to regulate the establishment of seed dormancy [99]. Seeds of the loss-of function mutant *myb96* exhibit a reduced dormancy and viviparous phenotypes are observed after harvesting siliques at long-green stage. Inversely, germination is delayed in activation-tagging *myb96-1D* seeds. In seedlings, expression of *NCED2*, *NCED5*, *NCED6* and *NCED9* genes is upregulated; however, MYB96 was shown to bind to *NCED2* and *NCED6* promoters only. MYB96 has also been reported to bind to *ABI4* promoter; nevertheless, it has been suggested to stimulate ABA biosynthesis in an ABI4-independent manner [100].

A recent genome-wide association study (GWAS) for seed coat color in soybean (*Glycine max*) revealed the existence of regulatory mechanisms at the protein level. The *G* gene that is responsible for seed green color has been shown to encode a CAAX amino-terminal protein protease and control seed dormancy in soybean [101]. *G* orthologs were selected upon domestication in several other crop species and *G* gene has a similar function in Arabidopsis. Interestingly, the encoded protein interacts with two ABA biosynthesis enzymes NCED3 and phytoene synthase, and positively affects ABA accumulation. Lower hormone levels and dormancy depth in mutant seeds suggest that G may have an essential contribution in dormancy induction.

Chromatin modification plays an essential role in transcriptional regulation of many physiological processes. In a recent study, Chen et al. reported that histone demethylation by the methyltransferase RELATIVE OF EARLY FLOWERING 6 (REF6) reduces seed dormancy by inducing ABA catabolism in seeds [102]. REF6 catalyzes the lysine (K27) demethylation of histone H3 (H3K27m3), of which methylation is associated with gene silencing. REF6 directly binds to *CYP707A1* and *A3* genes, but not *CYP707A2*. In developing siliques, REF6 reduces the level of H3K27 methylation levels in both genes. In accordance, in the *ref6* mutant, *CYP707A1* and *CYP707A3* gene expression is decreased during seed development and also during germination, this lower expression is correlated with enhanced ABA and dormancy levels in freshly-harvested seeds. Another study previously showed that the two Arabidopsis homologs of the human LYSINE SPECIFIC DEMETHYLASE 1, LDL1 and LDL2, which reduce the H3K4 methylation levels, act redundantly in the repression of seed dormancy [103]. LDL1 and LDL2 are highly expressed during early silique development and the increased seed dormancy in *ldl1 ldl2* double mutant is associated with an upregulation of *ABA2* transcripts at this stage. The increased expression of *ABI3* and *DOG1* signaling factors, which is observed in mutant seeds at later maturation stages may also contribute to dormancy enhancement. Other chromatin modifications have been described to affect ABA metabolism genes and seed dormancy. The HUB1/RDO4 E3 ubiquitin ligase is involved in histone H2B ubiquitination, which is associated with active gene transcription. HUB1 loss-of-function has been shown to reduce dormancy and be correlated with decreased expression of *NCED9* and *CYP707A2* in freshly-harvested mutant seeds compared to wild-type [104]. Histone deacetylation mediated by two histone deacetylase-binding factors, SWI-INDEPENDENT3 (SIN3)-LIKE1 (SNL1) and SNL2, has also been reported to influence seed dormancy [105]. Expression of *SLN1* and *SLN2* genes increase during seed development and these two genes likely have a redundant function in seed dormancy induction, since double mutant seeds exhibits lower dormancy levels than those of simple mutants. Furthermore, the reduced ABA content in mutant seeds correlates with an upregulation of *CYP707A1* and *CYP707A2* expression and, in accordance, both genes exhibit increased H3K9/18 acetylation levels. SNL1 and SNL2, which also act on ethylene pathway, would regulate seed dormancy and germination by modulating the antagonism between ABA and ethylene.

DNA methylation is an additional epigenetic modification that can repress gene transcription. In tomato, an epimutation that causes an hypermethylation in the promoter of the *COLORLESS NON-RIPENING* (*CNR*) gene prevents fruit ripening and coloring. The *Cnr* mutant possesses a hypermethylated epigenome whose maintenance requires a number of methylation enzymes, including methyltransferase1 (SlMET1). Interestingly, Yao et al. observed a correlation between *SlMET1* silencing and seed vivipary in *Cnr* fruits [106]. A decrease in ABA concentration was also reported together with a reduced accumulation of *SlNCED* and *SlZ-ISO* transcripts and a differential methylation of promoter regions, suggesting that an epiregulation of these genes prevents vivipary during fruit development.

## 4. ABA Metabolism during Dormancy Release and Germination

### 4.1. Importance of ABA Hydroxylation in the Control of Germination

Dormancy of mature dry seeds is progressively released upon dry storage, also called after-ripening, however, molecular mechanisms involved remain elusive. Limited changes in gene expression between dormant and non-dormant dry seeds have been observed, probably due to the lack of available free water for enzymatic reactions in a fully dry state, while passive and non-enzymatic reaction events such as oxidation and Amadori–Maillard reactions, which occur during after-ripening, have been suggested to be related with dormancy release [6,107]. Moreover, differences in dormancy depth are generally not well correlated with ABA contents in after-ripened seeds compared to dry dormant seeds. In contrast, variations in ABA levels in imbibed seeds appear clearly correlated with seed dormancy. A decrease in ABA levels is observed in both dormant and non-dormant seeds, however dormant seeds maintain higher ABA levels, as observed in Arabidopsis and barley [108,109]. In Arabidopsis, expression of *CYP707A2* is rapidly induced upon seed imbibition and plays a major role in the rapid decrease in ABA levels during early seed imbibition [53,55]. Similarly, barley *HvCYP707A1* is highly expressed during seed germination and increased expression is followed by a rapid decrease in ABA content [73]. Moreover, compared to after-ripened seeds, both Arabidopsis and barley dormant seeds retain a higher concentration of ABA and, in accordance, exhibit lower *CYP707A* transcript levels [110]. In parasitic plants, such as Orobanchaeceae, seed germination requires the presence of host-derived strigolactones as signal for germination. The stimulation of germination is correlated with an early upregulation of *CYP707A* gene expression in seeds upon imbibition in presence of GR24, a strigolactone analog [111,112].

Despite ABA catabolism appears to be a major contribution to regulation of ABA levels and dormancy in imbibed seeds, several lines of evidence suggest that ABA biosynthesis contributes to dormancy maintenance. Indeed, treatment with ABA biosynthesis inhibitors was shown to promote germination of dormant Arabidopsis seeds [109,113]. However, no differential expression of Arabidopsis *NCED* genes has been observed between dormant and non-dormant imbibed seeds, whereas in barley and *Brachypodium distachyon* respectively, *HvNCED1* and *BdNCED1* expression is increased in dormant embryos [110,114].

Beside activation of gene transcription, messenger RNA degradation has been suggested to efficiently act in the fine-tuning of gene expression during seed germination. Two 5′-3′ RNA decay mutants, respectively impaired in the 5′ to 3′ exoribonuclease XRN4 and the component of the decapping complex VARICOSE (VCS) display opposite dormancy and germination phenotypes [115]. In imbibed mutant seeds, germination phenotypes are well correlated with the modifications in the expression of the ABA and GA metabolism genes. In the dormant *xrn4* mutant, an increase in *NCED6* and *NCED9* and a decrease in *CYP707A2* transcript abundance are observed, and opposite expression patterns are observed in the nondormant *vcs* mutant. Moreover, these mutants also display an antagonistic regulation of ABA and GA metabolism genes, suggesting that RNA stability may contribute to the control of ABA/GA balance and dormancy maintenance.

Several studies have highlighted the importance of ABA produced in the endosperm in dormancy maintenance during imbibition [116,117]. A seed coat bedding assay showed that the growth of Arabidopsis dissected embryos is prevented when they are cultured on a layer of dormant seed coats. Indeed, unlike nondormant coats, dormant coats actively produce and release ABA to repress embryo germination [117]. Dissected seed coats are composed of a maternal testa, which in mature seeds is composed of dead dry tissues, and a single layer of endosperm, which is a living tissue that can be a source of ABA for the embryo. Four ATP-binding cassette (ABC) transporters, AtABCG25, AtABCG30, AtABCG31 and AtABCG40 have been reported to mediate ABA transport in imbibed seeds [118]. AtABCG25 and AtABCG31 function in ABA export from the endosperm and AtABCG30 and AtABCG40 in its import into the embryo. Mutant analysis also suggested that these transporters have a role in the hormonal control in seed dormancy and germination. In Arabidopsis, the embryo radicle has been proposed as a decision-making center in dormant seeds, in which ABA and GA metabolism and signaling proteins are expressed in distinct tissues. The decision to germinate or remain dormant, in response to environmental signals, would rely on dynamic regulation of the abundance of both hormones through complex feedback series involving hormone transporters [119].

### 4.2. Influence of Light, Temperature and Nitrate on Germination

Seeds monitor their environment to germinate under suitable conditions for progeny survival and hormone levels have an essential role in the integration of seed responses to these exogenous signals. Light, temperature and nitrate, together with soil moisture, have been shown to have a prominent influence. Light and temperature responses rely on the antagonistic regulation of the ABA and GA metabolism and signaling pathways, including the opposite regulation of *NCED* and *CYP707A* genes. In dicot seeds, white light activates germination by the perception R/FR wavelengths by the phytochrome family of photoreceptors [120]. In Arabidopsis seeds, an R light pulse has been shown to reduce ABA levels and decrease *ZEP*, *NCED6* and *AtNCED9* gene expression, whereas expression of *CYP707A2* was increased [121]. In lettuce (*Lactuca sativa*), ABA levels are controlled by the downregulation of *LsNCED2* and *LsNCED4* expression and upregulation of *LsABA8ox4* [122]. In contrast, in numerous monocot species and particularly in cereals, white light inhibits germination and blue light perception by cryptochromes has a similar effect. Crop species, such as barley and wheat, lack the R/FR light response, which has been presumably lost upon domestication [114]. In barley, continuous white light promotes dormancy of freshly-harvested dormant grains, whereas imbibition in the dark breaks dormancy. Germination inhibition by white light is correlated with increased ABA levels and *HvNCED1* expression in embryos; however, light or dark had no or little effect on *HvNCED2* and *HvABA8′OH1* expression. Blue light mimics the effect of white light on ABA and *HvNCED1*, in contrast to R and FR light that has no effect [123]. Furthermore, a long exposure under blue light at low temperature (10 °C) results in a progressive inability to germinate in the dark, considered as secondary dormancy [124]. In these imbibition conditions, blue-light inhibition was associated with increased ABA levels in embryos and a strong upregulation of both *HvNCED1* and *HvNCED2*.

Together with light, the ability of seeds to sense temperature is ecologically important for the seasonal timing of germination. In crop species, hot temperatures resulting from global warming are regarded as a major threat affecting seed germination. The seeds of many winter annual plants, such as Arabidopsis, germinate in the cool temperatures of autumn, but not in the hot temperatures of summer. In Arabidopsis, high temperature inhibits germination by altering the ABA/GA balance. Increased ABA accumulation results from the activation of ABA biosynthesis genes, including *ZEP*, *NCED2*, *NCED5* and *NCED9* and repression of ABA catabolic genes *CYP707A1-3* [125]. Most lettuce varieties exhibit seed thermoinhibition and *LsNCED4* has been identified by QTL analysis as the causal gene. When imbibed at 35 °C, seeds from a thermosensitive cultivar accumulate high ABA levels compared to seeds of the thermotolerant *Lactuca serriola* accession. Furthermore, in thermosensitive accessions, germination inhibition is correlated with an increased *LsNCED4* expression in imbibed seeds, which is not observed in thermotolerant accessions [126]. In wheat, germination inhibition at supraoptimal (35 °C) and suboptimal (4 °C) temperatures is better correlated with the expression of *TaNCEDs* than *TaCYP707As,* suggesting that ABA biosynthesis has the major role in temperature responses [127].

As previously mentioned, when Arabidopsis mother plants are fed with high nitrate, endogenous nitrate in developing seeds activates ABA catabolism by CYP707A2, resulting in reduced ABA levels and low dormancy, whereas low nitrate nutrition has the opposite effect [80,81,83]. Upon imbibition, exogenously applied nitrate also alleviates seed dormancy and stimulates germination by reducing ABA levels [82]. In Arabidopsis and the hedge mustard *Sisymbrium officinale*, low concentrations of nitrate (0.1 mM) have been shown to promote seed germination independently of its assimilation, suggesting that nitrate itself acts as a signal. In particular, nitrate is able to stimulate germination of the Arabidopsis nitrate reductase-deficient mutant *nia1nia2* mutant [80]. In both species, nitrate has been reported to reduce ABA levels by upregulating *CYP707A2* expression and, in accordance, nitrate fails to decrease the ABA content in the Arabidopsis *cyp707a2* mutants [80,83,128]. Altogether, nitrate negatively regulates both the induction of primary dormancy during seed development and its maintenance during germination, by activating ABA catabolism at both developmental stages.

### 4.3. Regulatory Factors of ABA Metabolism in Imbibed Seeds

Germination is regulated by the antagonistic ABA/GA balance and ABA catabolism upon imbibition precedes GA synthesis and activation of germination. Several transcription factors have been shown to oppositely regulate ABA and GA metabolism pathways. Among them, ABI4 has been described above to act as a positive regulator of dormancy induction during seed development and a repressor of ABA catabolism through its binding to the *CYP707A1* and *CYP707A2* promoters. During germination, ABI4 regulates both ABA synthesis and catabolism. In *abi4* imbibed seeds, a downregulation of *NCED* genes and an upregulation of *CYP707A* genes is observed, *CYP707A2* transcripts being the most differentially accumulated [97]. At seedling stage, ABI4 has been shown to activate *NCED6* and *GA2ox7*, a GA-inactivating gene, by direct binding to promoters. Moreover, ABA and GA are able to antagonistically modify ABI4 expression and stability, suggesting the existence of regulatory loops [95]. However, the occurrence of such regulatory processes in germinating seeds remains to be confirmed. Interestingly, the ABA-responsive transcription factor MYB96, highly expressed in embryo, regulates seed germination by controlling the expression of *ABI4* in Arabidopsis [129]. In rice, the transcription factor OsAP2-39 which, like ABI4, belongs to the APETALA2-domain family has been shown to be involved in the regulation of the ABA/GA balance and to bind to *OsNCED1* promoter. Furthermore, *OsAP2-39* overexpression upregulates *OsNCED1* transcript and ABA levels in leaves; however, again evidence for a regulatory role in germinating seeds is still lacking [130].

**Figure 4:**
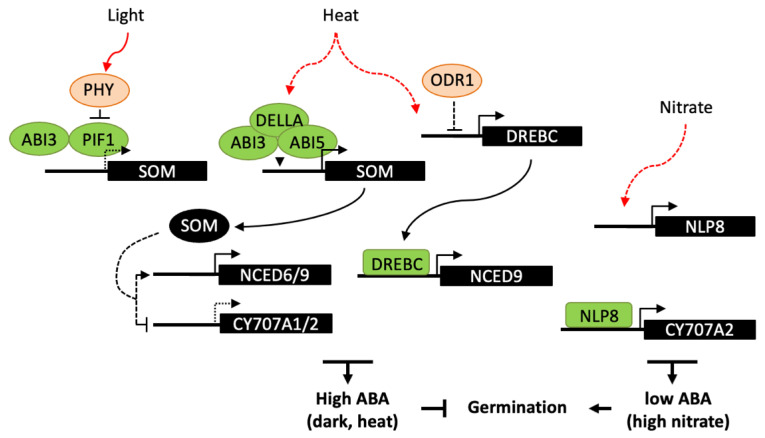
Transcriptional regulation of ABA metabolism in germination responses to light, high temperature and nitrate. Light-activated phytochromes interact with PIF1 and promote its degradation. In the dark, PIF1 interacts with ABI3 and both bind to the promoter of *SOM* gene, which indirectly regulates ABA metabolism genes, resulting in an increase in ABA levels and inhibition of germination. Similarly, SOM is also indirectly involved in germination thermoinhibition, ABI3 and ABI5 form a complex with DELLA proteins and bind to the *SOM* promoter. In response to heat, DREBC has been shown to directly binds to *NCED9* promoter and upregulates ABA accumulation. Moreover *DREBC* expression would be indirectly subject to a negative regulation by ODR1, which role in temperature response has not been investigated. Nitrate promotes germination through NLP8 binding to *CYP707A2* promoter and activation of its expression, thus reducing ABA levels upon imbibition. Dashed lines indicate either an indirect effect of the signaling factor, or a reduced expression of the downstream gene.

A number of transcriptional regulators have been shown to be involved in germination responses to light, including ABA and GA signaling factors [120,131]. In Arabidopsis, phytochrome B (PHYB) is the main photoreceptor in germination activation by R light and reversion by FR light, whereas PHYA promotes germination responses in continuous FR light and at very low fluence rates. Light-activated phytochromes interact with the basic helix–loop–helix (bHLH) transcription factor PHYTOCHROME-INTERACTING FACTOR 1 (PIF1) and promote its degradation by the 26S proteasome (Figure 4). PIF1 directly regulates the expression of several downstream genes, including the C3H-type zinc finger protein SOMNUS (SOM), which contributes to the inhibition of seed germination through the modulation the GA and ABA metabolism genes, including the activation of the ABA biosynthesis genes, *ABA1*, *NCED6* and *NCED9* and the inhibition of the catabolic gene *CYP707A2* [132]. Like PIF1, ABI3 is a positive regulator of SOM expression by direct binding to RY motifs in its promoter and, at the protein level, ABI3 and PIF1 interact to collaboratively activate SOM expression in imbibed seeds [133]. In maturing seeds, ABI3 has also been shown to upregulate SOM expression but independently of PIF1, suggesting that other transcription factors may be involved at this stage. Light responses in Arabidopsis seeds have been shown to be spatially-controlled [134]. FR light inactivates PHYB in the endosperm whereas it activates PHYA in the embryo. PIF1 stabilization in the endosperm results in the downstream repression of GA synthesis genes and activation of *NCED6* and *NCED9;* ABA released from the endosperm would then counteract PHYA signaling in the embryo and repress germination.

Downstream factors in the light signaling network controlling seed germination remain to identify, in particular the direct regulators of ABA biosynthesis or catabolism genes. Recently, ABI4 has been reported to promote PHYA-dependent seed germination [135]. A 48-h FR light treatment results in the activation of PHYA and deactivation of PHYB. Under these conditions, *abi4* mutant seeds germinated at lower rates and expression of *NCED6* and *NCED9* genes was upregulated. Therefore, PHYA has been suggested to promote *ABI4* expression, through the action of PIF1, then ABI4 would in turn repress ABA biosynthesis and activate germination. The repressive function of ABI4 on ABA biosynthesis under FR light contrasts with the conclusions of the other study detailed above [97], which described ABI4 as a repressor of germination and activator of ABA biosynthesis. Since observations have been made in very different experimental conditions, understanding of ABI4 regulatory functions would require further study.

The control of plant growth by light and temperature has been shown to share a large set of common regulators, including phytochromes and PIF proteins [136]. However, in seeds, molecular mechanisms involved in temperature sensing to activate or suppress germination remain elusive. In Arabidopsis seeds, the SOM gene appears to integrate both light and high-temperature signals. ABI3 interacts with either PIF1 or the bZIP transcription factor ABI5, in the dark or at high temperature respectively, resulting in repression of germination. At high temperature, both ABI3 and ABI5 form a complex with the GA-signalling proteins DELLA and bind to the SOM promoter to activate its expression [137]. Moreover, SOM may participate to a positive feedback loop with ABI3, ABI5, and DELLAs, through the activation ABA biosynthesis and inhibition of GA biosynthesis. Besides thermoinhibition, high temperature can inhibit germination by the induction of secondary dormancy, which does not allow seeds to resume germination for a while even after a return to favorable temperatures [138]. PHYD has been shown to modulate secondary dormancy and could act by controlling PIF1 degradation, which in turn may impact on expression on ABA and GA metabolism genes and favor ABA accumulation [139]. High temperature has also been reported to enhance the expression of *FUS3* [140]. *FUS3* overexpression results in delayed seed germination at high temperature, while *fus3* mutants are more tolerant than wild type. FUS3 activation by heat stress is correlated with the antagonistic regulation of ABA and GA metabolism and signaling genes, in particular with an upregulation of *NCED5* and *NCED9*, however the exact role of FUS3 is still unknown and might be indirect. A more direct regulatory function has been reported for the transcription factor DREBC, since it is able to bind *NCED9* promoter and its constitutive overexpression delays germination at supraoptimal temperature (33 °C) and increase seed ABA content [94].

In Arabidopsis, the nitrate transporter NRT1.1/CHL1 has been shown to have a dual function, acting as both a nitrate sensor and transporter, and to induce primary nitrate response independent of its function in nitrate transport [82]. Downstream of the nitrate sensor, NIN-like proteins (NLP) constitute a family of transcription factors that are involved in nitrate responses. In seeds, NLP8 is the most abundantly expressed NLP and, in good correlation, nitrate fails to promote germination of *nlp8* seeds in response to nitrate. NLP8 directly binds to the nitrate response element (NRE) in the *CYP707A2* promoter region and induces gene expression, suggesting a key function for NLP8 in nitrate signaling for the control of germination. Furthermore, since *nrt1.1*/*chl1* mutant exhibits a much milder germination insensitivity to nitrate than *nlp8*, NRT1.1/CHL1 may have a minor role in seed germination and other nitrate sensors may be involved upstream of NLP8 [141]. A number of other regulators upstream of ABI and SOMNUS have been described [5], which could have a downstream effect on ABA accumulation and gene expression. Furthermore, ODR1 which functions downstream of ABI3 in seed dormancy regulation [91] may possibly have a role in germination responses to exogenous signals.

## 5. Natural Variations of Genes Affecting ABA Metabolism in Seeds

### 5.1. Relationship between ABA Levels and Germination in Natural Lines

Natural inbred lines or strains of plants have been generated from wild ancestral plants under both natural and human selection. They exhibit a large variety of phenotypes in terms of shape, growth, or physiological response to the environment, and are valuable resources for crop improvement, as well as being successfully employed to identify the genes and the allelic diversity that are causative for the phenotypic differences [142,143,144,145]. In Arabidopsis, more than 1000 natural inbred lines have been collected from a range of ecologically diverse habitats [146,147] and were often called “ecotypes”. However, as it does not conform strictly to its ecological definition, they are now referred to as “accessions”, which has been often used to assign a unique identifier for a plant genotype of a species collected at a specific location [148,149].

It has been reported that levels of endogenous ABA in imbibed seeds differ between the natural lines or accessions of different plant species, being associated with germination phenotypes. For instance, in Arabidopsis seeds, ABA content decreased at 6 h after the onset of imbibition; however, the reduction was less in the deeply-dormant accession from Cape Verde Islands (Cvi) than in the weakly-dormant accession Columbia (Col) [150]. In wheat, freshly-harvested seeds from varieties Gifu and OS38 both maintained their dormancy when imbibed at 20 °C, with no significant changes in the endogenous amount of ABA. After imbibition at low temperature (15 °C), Gifu seeds became less dormant and germinated but OS38 did not, in good correlation with ABA level decrease in Gifu and increase in OS38 [151]. In lettuce, the accession Salinas showed an inability to germinate at a high temperature, while the accession UC96US23 was thermoinhibition-resistant; moreover, the ABA levels in imbibed seeds in the former accession were almost 5-fold greater than those in the latter accession [152]. The link between seed ABA levels and germination ability in natural lines suggests that natural variations in ABA metabolism genes may affect seed germination phenotypes. Again, the focus remains on genes involved in the ABA metabolism pathway, downstream of zeaxanthin. Thus, we summarize here the natural variations in both biosynthesis and catabolism genes and highlight their effect on ABA levels in seeds and germination-related phenotypes (Table 1), including also carotenoid content and seed color that often affect ABA biosynthesis and seed germination [153,154,155].

### 5.2. Natural Variations of ABA Metabolism Genes Associated with Germination-Related Phenotypes

Natural variations in *ZEP* genes have been reported in GWAS for carotenoid content rather than ABA levels in seeds. In Arabidopsis, GWAS for the ratio of violaxanthin to antheraxanthin (V/A) in mature dry seeds, followed by haplotype analysis, identified three haploblocks with contrasting haplotypes located near the *ZEP* genetic region that significantly affected the V/A ratio. The most significant haplotype is HB4a, which is located at the 3′ intergenic region of *ZEP*. This was corroborated by a quantitative trait locus (QTL) analysis with two parental accessions having contrasting HB4a haplotypes, Col-0 and Blanes-1 (Bla-1), which showed that the *ZEP* gene is within the QTL region significantly associated with the V/A trait. *ZEP* mRNAs were induced during mid-seed development, and the haplotype HB4a was shown to be correlated with modifications in transcript level and V/A content between accessions [156]. Similarly, single nucleotide polymorphisms (SNPs) within and proximal to *ZEP1* gene in maize were detected by GWAS for kernel carotenoid composition and kernel color, respectively [157,158]. SNPs in *ZEP1* associated with zeaxanthin levels in grain were also identified by GWAS in sorghum (*Sorghum bicolor*) [159]. A possible effect of natural variations in maize *ZEP* on ABA levels was reported by QTL analyses for kernel desiccation and ABA content during seed maturation. A colocation of *ZEP1* gene was found with both QTLs; however, the *ZEP1* mRNA levels in parental lines were not clearly correlated with the ABA contents [160]. Natural variation of *ABA4* and *NXD1,* of which mutations in Arabidopsis and tomato affect synthesis of neoxanthin and violaxanthin isomers and germination in seeds has also not been reported so far.

In terms of the effects of natural variations in *NCED* genes on seed germination, resistance to thermoinhibition in lettuce seeds has been studied in detail. As mentioned above, seeds of the UC96US23 accession have a lower ABA endogenous content than Salinas seeds and exhibit a thermoinhibition-resistant phenotype [152]. QTL analysis for the alleviation of thermoinhibition, with near-isogenic lines (NILs) derived from the two accessions, identified *LsNCED4* as a most promising candidate for a responsive gene [161]. Transgenic expression of Salinas *LsNCED4* driven by its native promoter in UC96US23 seeds resulted in thermoinhibition, with increased levels of endogenous ABA compared to wild-type UC96US23 seeds. *LsNCED4* coding regions from both UC96US23 and Salinas could rescue the thermotolerant phenotype of Arabidopsis *nced6-1 nced9-1* double mutant, indicating that both *LsNCED4* alleles of UC96US23 and Salinas encode functional NCED proteins. Nineteen SNPs on the promoter regions of *LsNCED4* were, however, conserved within thermotolerant or thermosensitive accession groups and the mRNAs expression patterns were associated with the two different groups. After imbibition at high temperature, *LsNCED4* transcripts declined to low levels in thermotolerant accessions, whereas levels were maintained in thermosensitive accessions. Variations in promoter sequences have been suggested to induce changes in binding specificities for trans-acting factors controlling *LsNCED4* expression [126]. In rice, *nced3* mutants germinated earlier than wild type [162]. In the cultivar PA64s mature embryos, *NCED3* expression was 3.9-fold higher than in the cultivar 9311, which was consistent with stronger dormancy and remarkably higher ABA levels in PA64s dry seeds, as compared to 9311. A comparison of *NCED3* sequences in both cultivars inferred SNPs and insertions/deletions (Indels) within the gene. A low similarity between 9311 and PA64 was found in regulatory sequences (promoter and UTRs), as well as potential amino acid changes in the coding regions [163], suggesting that these variations in rice *NCED3* may be involved in regulation of dormancy. Recently, it has been reported that natural variation of *NCED3* in Arabidopsis affects the accumulation of ABA in seedlings under low water potential stress. The ABA accumulation in the accession Shahdara (Sha) was less than that in the accession Landsberg *erecta* (L*er*). A QTL analysis for the ABA levels with recombinant inbred lines (RILs) derived from the two accessions revealed the major-effect QTL for the trait, which included *NCED3* as a candidate gene. Complementation experiments indicated that Sha has a reduced-function *NCED3* allele and has four amino acid substitutions within AtNCED3 compared with L*er*, although how these amino acid substitutions could alter AtNCED3 activity remain to be elucidated [164]. Interestingly, the QTL for ABA accumulation including *NCED3* colocalized with previously detected QTLs for speed of seed germination (faster germination of Sha than L*er*) and ability to germinate on salt- or ABA- containing media (higher germination rate in Sha than L*er*) [165], implying that *NCED3* natural variation may be involved in these differences through the regulation of ABA levels. Aiming to know ABA-mediated anthocyanin biosynthesis and pericarp coloration, natural variation of rice *NCED2* has been recently analyzed [166]. No clear link was observed between the pericarp color and a single nucleotide substitution from C to T in the *NCED2* gene that causes a valine to isoleucine change in the protein. The same substitution to isoleucine in NCED2 was however, dominant in many upland rice varieties and these rice varieties exhibited a higher level of ABA in leaves and were more tolerant to drought [167]. Altogether, future research is still needed concerning the relationship between endogenous ABA levels, seed phenotypes and the natural variations of *NCEDs*.

For steps downstream of xanthoxin synthesis, little information is available on the natural variation of these biosynthesis genes that directly affect seed ABA content. Nevertheless, in wheat grain, yellow pigment content and yellow index (color intensity) were significantly associated with SNPs in the coding sequences of *ABA2* and *AO3* [168]. Moreover, *AO3* mRNAs were more abundant in seeds of the cultivar Ciccio than in those of the cultivar Svevo that have a higher carotenoid content, indicating that the allele present in Ciccio is associated with a more active carotenoid degradation, although no polymorphisms were observed in the promoter regions between the two cultivars [169]. In soybean, nine possible candidate genes were identified by GWAS for seed germination under salt stress, in which a candidate gene is homologous to *ABA3* and located near the SNP marker [170].

Among genes in ABA catabolic pathways, natural variations in *CYP707A/ABA8′OH*, may have a link to ABA amount and germination ability in seeds. For instance, mRNA expression of the gene *CYP707A5* in rice was 3.5-fold lower in the cultivar PA64s than in the cultivar 9311, and coincided with levels of dormancy and ABA in the two cultivars. SNPs and Indels in regulatory and coding regions of *CYP707A5* may induce these differences between the two cultivars [163]. In wheat, *ABA8′OH-2* has also been mapped near to a major QTL for seed dormancy. Five amino acid residue substitutions in ABA8′OH-2 were inferred by comparing allele sequences between parental accessions for the QTL. One of the substitutions occurs in a highly conserved amino acid residue in *CYP707A* family, implying that the substitution may impact on the enzymatic activity of ABA8′OH-2 [171].

**Table 1:** Natural variations of ABA biosynthesis and catabolic genes that affect germination-related phenotypes.

Gene	Species	Types of Genetic Variation	Involvement in ABA Metabolism and Phenotype	Ref.
*ZEP*	Arabidopsis	Intergenic SNPs/QTL region	Affecting mRNA levels of *ZEP* and ratio of violaxanthin to antheraxanthin	[156]
*ZEP1*	Maize	SNPs in coding region	Associated with carotenoid composition	[157]
		Intergenic SNPs	Associated with kernel color	[158]
		QTL regions	Colocating with QTLs for kernel desiccation and ABA content	[160]
*ZEP*	Sorghum	SNPs inside the gene	Associated with zeaxanthin levels	[159]
*NCED4*	Lettuce	SNPs in promoter	Affecting mRNA levels of *NCED4,* ABA levels and thermoinhibition	[126]
*NCED3*	Rice	Low similarity of regulatory region SNPs and Indels in coding region	Higher expression at mRNA level in variety seeds with higher ABA level and stronger dormancy	[163]
*NCED2*		Nonsynonymous SNPs	Affecting ABA levels in leaves and tolerance to drought but not clearly associated with pericarp color	[166] [167]
*NCED3*	Arabidopsis	QTL region/Nonsynonymous substitutions	Affecting ABA accumulation in seedling under low water potential stress	[164]
		QTL region	Colocating with QTLs for germination speed and ability on media containing salt or ABA	[165]
*ABA2* *AO3*	Wheat	SNPs in coding region	Associated with yellow pigment content and yellow index of grain	[168]
*AO3*		No clear polymorphisms in promoter	Highly expressed at the mRNA level in variety seeds with lower carotenoid content	[169]
*ABA3*	Soybean	Intergenic SNPs	Associated with seed germination under salt stress	[170]
*CYP707A5*	Rice	SNPs and Indels in regulatory and coding region	Lower expression at mRNA level in variety seeds with higher ABA level and stronger dormancy	[163]
*ABA8′OH-2*	Wheat	QTL region/Nonsynonymous substitutions	Colocating with QTL for seed dormancy	[171]

### 5.3. Perspectives for Understanding Natural Variations That Underlie ABA Metabolism in Seeds

Although several studies have reported altered ABA levels or germination in seeds of natural variants, our current knowledge regarding the causative natural variations of genes for ABA metabolism remains fragmentary. Furthermore, to date, no genetic screen specifically for ABA levels in seeds has been performed, although such an approach could be interesting to develop. Aiming to understand drought-induced ABA accumulation in natural accessions, GWAS for ABA levels in seedlings under low water potential was performed in Arabidopsis [172]. The genetic regions containing clusters of ABA-associated SNPs were validated by reverse genetic analyses and six effectors of ABA accumulation such as plasma membrane protein and RING-U box protein were identified. Interestingly, none of these six genes were previously reported to affect ABA or abiotic stress response. Further insight into the natural variations affecting ABA metabolism in seeds may be gained by a similar strategy combining GWAS for seed ABA levels and reverse genetics.

GWAS have succeeded to expand the catalog of variants underlying human diseases, however, as many as 90% of the variants fall within non-coding regions having unknown functional importance [173]. Similarly, many of natural variations in genes summarized in this review are in intergenic or regulatory regions rather than in the coding regions implying that, in seeds of natural variant lines, their function is often regulated at the transcript level rather than enzyme activity. Recently, the natural variations in regulatory regions that control gene expression response to drought stress in maize were revealed by GWAS focusing on transcriptome in leaves. Ninety-seven genes were prioritized in relation to their association with drought tolerance due to their variation in expression and, among the candidates, *Zm00001d051554* (*abh2*) encoding ABA 8′-hydroxylase was verified to play a negative role in the drought tolerance [174]. Therefore, transcriptome-wide association study would also be a promising approach to understand natural variations of genes related to ABA metabolism in seeds.

Natural variations of ABA metabolism genes may play an important role in adapting plants to various environments within a species. Nonetheless, it should be noted that the identified genetic variations do not always explain the local adaptation within the species because in a typical approach, a subset of genotyped lines is grown together under conditions defined the “common garden”, without considering the complex natural environment in which each line inhabits [175,176]. Furthermore, differences in endogenous levels of ABA cannot always explain germination variety between natural lines, and other processes such as ABA perception and signal transduction are also important for the regulation for variety in seed dormancy within a species. For instance, during seed development in 14 accessions of rice, pre-harvest sprouting (PHS)-susceptible accessions maintained similar or even higher ABA levels compared with PHS-resistant accessions [177]. In addition, a number of genes identified in QTLs functioning in cereal PHS are involved in ABA signaling rather than metabolism, although many causal genes for this trait remain to be identified [178,179].

## 6. Conclusions

In recent years, our understanding of the regulation of ABA metabolism in seeds has been greatly improved and most enzymes of the ABA biosynthesis and catabolism pathways have been identified. Nevertheless, several key steps still remain obscure, such as the synthesis of the last carotenoid precursors of ABA or translocation mechanisms of precursors within the chloroplast and from chloroplast to cytosol. Facets of the molecular factors and mechanisms that regulate ABA synthesis and degradation are also gradually being resolved. Interestingly, several novel regulators have been identified as directly acting on ABA metabolism genes in seeds. Some others have been characterized in vegetative tissues, of which function could be assessed in seeds. Regarding the factors that control the tissue localization of ABA, several ABA transporters have been identified but only a small number has been functionally characterized in seeds [16]. ABA movement within seed tissues, notably between endosperm and embryo, and from mother plant tissues to seeds would be particularly important to decipher for a better understanding of the role of these distinct ABA pools in signaling pathways that control dormancy induction and germination, in response to environmental conditions such as light, temperature and nitrate. It would also require the development of efficient technologies to precisely assess local ABA concentrations in seed tissues. Besides strategies such as engineering ABA receptors or developing ABA receptor agonists [180], natural variants implicated in ABA metabolism or signaling could be valuable materials for improvement of important traits for crop cultivation such as stress tolerance and germination characteristics. Since, in general, mutations in key genes of plant hormone metabolism/signaling affect overall plant growth, the discovery of natural variations that fine-tune ABA action in a specific spatiotemporal manner during vegetative or reproductive development and in response to stress will be important in the future. Identification of the variations that directly affect the expression of ABA metabolism-related genes may be prioritized for the analysis.
